# Association of Ambient Air Pollution with Increased Liver Enzymes in Korean Adults

**DOI:** 10.3390/ijerph16071213

**Published:** 2019-04-04

**Authors:** Hyun-Jin Kim, Jin-young Min, Yong-Seok Seo, Kyoung-bok Min

**Affiliations:** 1 National Cancer Control Institute, National Cancer Center, Goyang 10408, Korea; hyunjin@ncc.re.kr; 2 Institute of Health and Environment, Seoul National University, Seoul 08826, Korea; yaemin00@snu.ac.kr; 3 Disaster Management Research Center, Seoul 05402, Korea; yss523@dmrc.kr; 4 Department of Preventive Medicine, College of Medicine, Seoul National University, Seoul 03080, Korea

**Keywords:** ambient air pollution, association, liver enzymes, Korean adults

## Abstract

An association between exposure to air pollution and liver enzymes in certain areas or older people has been reported in the literature; however, it cannot be generalized to the general population. We investigated the association between air pollution, liver enzyme levels, and alcohol consumption using nationwide data of South Korean adults. Air pollutants included particulate matter with an aerodynamic diameter ≤10 µm (PM_10_), nitrogen dioxide (NO_2_), sulfur dioxide (SO_2_), and carbon monoxide (CO). Liver enzymes included alanine aminotransferase (ALT) and aspartate aminotransferase (AST). Exposure to air pollutants were significantly associated with elevation of log ALT and log AST, especially increases from 0.0073 IU/L (95% confidence interval (CI) = 0.0042, 0.0104) to 0.0251 IU/L (95% CI = 0.0132, 0.0371) per interquartile range (IQR) increase of each pollutant (all pollutants: *p* < 0.001). Association of the liver enzymes with PM_10_ (β (95% CI) = 0.0285 IU/L (0.0201, 0.0368) for log ALT; β (95% CI) = 0.0139 IU/L (0.0079, 0.0198) for log AST) and CO (β (95% CI) = 0.0247 IU/L (0.0182, 0.0311) for log ALT; β (95% CI) = 0.0164 IU/L (0.0118, 0.0210) for log AST) were only significant among drinkers. Our findings suggest that chronic exposure to PM_10_ and CO is a risk factor for liver enzymes increases among the general adult population who admitted to drinking alcohol.

## 1. Introduction

Ambient air pollution is a serious health problem. The World Health Organization (WHO) estimates the number of deaths per year by air pollution is approximately 3 million worldwide. Exposure to airborne pollutants has been associated with increased risk of, and premature death from, various diseases, including cardiovascular disease (CVD), respiratory disease, and certain cancers [1,2,3]. Ophthalmic conditions, such as dry eye syndrome, and even mental disorders have been associated with air pollution [4,5].

The pathological link between air pollution and disease has been attributed to oxidative stress [6] in CVD, neurodegenerative disease, lung disease, and cancer [7,8,9]. The liver, which plays a key role in maintaining metabolic homeostasis, is highly responsive to oxidative stress [8,10,11], and the potential negative effect of air pollution on the liver has previously been discussed. In 2013, Markevych et al. hypothesized that changes in liver enzyme levels may constitute a link between ambient air pollution and CVD [12]. This was supported by their finding that chronic exposure to airborne particulate matter with an aerodynamic diameter of ≤2.5 µm (PM_2.5_) was associated with changes in γ-glutamyl transferase (GGT) level. The authors emphasized the need for replication of the findings by additional investigations.

Alcohol consumption also increases the risk of liver disease [13,14]. A recent study on the elderly in Korea reported that short-term air pollution exposure contributed to increases in liver enzyme levels and that the effect was decreased by abstinence from alcohol [15]. The few studies that reported associations between short-term or chronic exposure to ambient air pollution and liver enzymes were limited regionally. Those that included the concomitant effect of alcohol consumption focused on short-term exposure and limited age groups, such as the elderly. Therefore, large-scale studies in a general adult population with nationwide data are required to generalize the results.

This study aimed to determine whether chronic exposure to air pollutants, including particulate matter with an aerodynamic diameter ≤10 µm (PM_10_), nitrogen dioxide (NO_2_), sulfur dioxide (SO_2_), and carbon monoxide (CO), is associated with liver enzyme levels using large-scale nationwide data in South Korea. We also assessed the modification effect of alcohol consumption on this association.

## 2. Materials and Methods

### 2.1. Study Sample

The subjects in this study were obtained from the Korean National Health and Nutrition Examination Survey (KNHANES). The KNHANES is a nationwide population-based survey designed to identify the health-related lifestyle, nutritional status, and health outcomes of Koreans. This survey, conducted by the Korean Centers for Disease Control and Prevention, considered a complex and multi-stage probability sampling method for the representation of data for the whole Korean population. First, the primary sample units (PSUs) are selected based on all census blocks or administrative districts. Each PSU consists of about 50 to 60 households. Second, the systematic sampling method was used to select 20 households in each PSU, except for foreign households or specific facilities such as prisons or military facilities. Lastly, all members (aged 1 year and over) in each selected household were considered as study participants. Approximately 10,000 individuals are sampled from 192 PSUs per year. This survey collected a lot of information about demographic variables, health-related behaviors, anthropometric measures, and clinical profiles [16]. Data from the 4th–6th KNHANES (2007–2015) were used for this study, and a total of 73,353 subjects were surveyed during this period. Of the total samples, we excluded individuals who did not meet the inclusion criteria of the study (i.e., (1) adults over 20 years old; (2) those that have information on their residential administrative area; (3) those whose liver enzyme levels were measured; and (4) those with accurate information about confounding factors). Therefore, a total of 36,151 adults were included in the final statistical analysis. This study was approved by the institutional review board of the Seoul National University Hospital Biomedical Research Institute.

### 2.2. Liver Enzyme Measurement

Elevated alanine aminotransferase (ALT) and aspartate aminotransferase (AST) levels are known as biochemical indicators of liver dysfunction, including liver damage [17]. These enzymes are also commonly used in liver function tests. Therefore, ALT and AST were the liver enzymes considered for this study. To analyze serum ALT and AST levels, laboratory measurements were performed after ≥8 h of fasting. Blood samples were collected in a serum separator tube. The collected samples were stored at 2–8 °C and analyzed within 24 h. A professional analytical agency carried out a biochemical analysis on blood samples from all subjects with a Hitachi Automatic Analyzer 7600 (Hitachi High-Technologies, Tokyo, Japan).

### 2.3. Air Pollution Exposure

To determine exposure to ambient air pollution, we obtained the atmospheric data (1 January 2007 to 31 December 2015) for 24 h concentrations of air pollutants, such as PM_10_, NO_2_, SO_2_ and CO, from the Ministry of the Environment of South Korea. These concentration values were measured at about 283 nationwide monitoring sites in South Korea. The concentration data measured at each monitoring station are centralized to the National Ambient air quality Monitoring Information System (NAMIS), which is based on the Internet and published online on the Air Korea website. To determine the effect of air pollutants on liver enzymes, we used the annual average concentrations of the administrative districts, including seven metropolitan and nine states, based on the above 24 h monitoring data [4,18,19]. Residential administration district codes and survey year were considered to link each individual to the annual average concentrations of ambient air pollution. Therefore, we assessed levels of air pollution using an alternative approach that assigned the same level of exposure to individuals living in the same administrative district. We finally excluded Jeju Island, which has considerable socio-cultural and environmental differences compared to other administrative regions.

### 2.4. Variables of Interest

To control the effects of confounders on association between air pollution and liver enzyme levels, we considered demographic information, health-related lifestyle or behaviors, and clinical variables [12,20,21]. The demographic data, including age, sex, education level, household income, and urbanity, were obtained from a questionnaire conducted by trained interviewers. Health-related lifestyles such as smoking status, alcohol drinking, moderate physical activity, and stress status were assessed using the questionnaire and classified as categorical variables as follows: Smoking status (current, former, or never smoker), alcohol consumption frequency per month (never, less than 1 time, 2 to 3 times, or more than 4 times), moderate activity (yes or no), and daily life stress (yes or no). To identify modifications to alcohol drinking status, we also divided alcohol drinking into two categories: Non-drinkers and drinkers. In addition, we included clinical variables such as body mass index, waist circumference, and disease history of total cholesterol, hypertension, diabetes, and cardiovascular disease. The anthropometric measurements, including height (m), weight (kg), and waist circumference (WC) (cm), were obtained, and the body mass index (BMI) was calculated by dividing the weight (kg) by the height^2^ (m^2^). Systolic blood pressure (SBP) and diastolic blood pressure (DBP) were measured three times, respectively, and the average values of the second and third measurements were used for this study. Hypertension was defined SBP ≥ 140 mm Hg or DBP ≥ 90 mm Hg, or those taking hypertensive medications for more than 20 days a month. We also obtained total cholesterol (TC) and fasting blood glucose (FBG) levels from blood analysis, and defined diabetic patients as having an FBG of 126 or more or taking an insulin injection or diabetic drugs. In addition, CVD was defined as having been diagnosed with a stroke or myocardial infarction.

### 2.5. Statistical Analysis

We checked the distribution of our liver enzymes phenotypes before analyses. Because ALT and AST followed a right-skewed distribution, their concentration values were converted to natural logarithms to approximate the normal distribution. Univariate analysis was performed to investigate the covariate variable for ALT or AST, and all significant variables were included as confounding factors. To identify the association between air pollution exposure and liver enzyme levels, we performed the multiple linear regression analyses with log-transformed phenotypes. The beta coefficients and 95% confidence intervals (CIs) for log ALT and log AST were estimated, and these estimates were scaled to the interquartile range (IQR) for each pollutant (9 μg/m^3^ for PM_10_, 11 ppb for NO_2_, 1 ppb for SO_2_, and 0.1 ppm for CO). We fitted the four statistical models via gradual adjustments: Crude (unadjusted for covariates); Model 1, adjusted for demographic variables including age, sex, education level, household income, and urbanity; Model 2, adjusted for demographic variables plus life style including smoking status, alcohol drinking, moderate physical activity, and stress status; Model 3, adjusted for demographic variables and health behaviors plus clinical variables including BMI, WC, TC, SBP, hypertension, diabetes, and CVD. In addition, stratified analyses by alcohol drinking status and alcohol consumption level were carried out, and the association results are presented after adjustment for full covariates (Model 3). All statistics were analyzed with SAS 9.3 (SAS Institute, Cary, NC, USA) and statistical significance was determined with a significance level of 0.05.

## 3. Results

The demographic characteristics, health behaviors, and clinical measurements of the 36,151 subjects who met all the inclusion criteria are shown in Table 1. The mean age of the subjects was 49.5 years. Fewer male (42.8%) than female (57.2%) were included; 33.9% of the participants had graduated from high school and 29% had a college or higher degree. The proportion of subjects living in rural areas (32.8%) was lower than that of subjects living in cities (67.2%). The proportion of current and former smokers was 21.4% and 19.9%, respectively. More than 70% (*n* = 25,921) drunk alcohol regularly, and approximately 10% (*n* = 2581) drank alcohol more than four times each month. Approximately 39.2% of the subjects said that they engaged in moderate physical activity and 26.8% said they were exposed to a usual stress level. The mean BMI was 23.7 kg/m^2^, and the mean WC was 81.3 cm. The mean SBP and DBP were 118.6 mm Hg and 76.2 mm Hg, respectively, and approximately 30% of the subjects were hypertensive. Approximately 10% of the subjects had diabetes and 4% had CVD. The mean ALT and AST were 21.7 IU/L and 22.5 IU/L, respectively. The demographic variables, health behaviors, and clinical measurements were significantly associated with log ALT or log AST (all *p* < 0.05)

Table 2 demonstrates the mean and median values of four air pollutants, PM_10_, NO_2_, SO_2_, and CO, in the urban and rural regions. The median PM_10_, NO_2_, SO_2_, and CO concentrations were 50.7 µg/m^3^, 25.1 ppb, 5.4 ppb, and 0.5 ppm, respectively. Their IQRs were 9 µg/m^3^, 11 ppb, 1 ppb, and 0.1 ppm, respectively. The average concentrations of all air pollutants were higher in urban areas than in rural areas.

The results showing the association between ambient air pollutants and liver enzymes, specifically ALT and AST, are shown in Table 3. In the crude Model, except NO_2_, all air pollutants were significantly associated with elevated log ALT levels (all *p* < 0.0001). The results were similar for Model 1, adjusted for demographic variables, and for Model 2, adjusted for demographic variables and health-related behaviors. After adjusting for all covariates, including clinical outcomes, Model 3 revealed that all four air pollutants were significantly associated with log ALT levels (all *p* < 0.0001). In the crude model, exposure to SO_2_ and CO was associated with increases in log AST levels. The IQR increase in SO_2_ (1 ppb) was associated with a 0.0057 IU/L (95% CI = 0.0023, 0.0091) increase in the log ALT level, and 0.1 ppm increase in CO was associated with a 0.0066 IU/L (95% CI = 0.0025, 0.0107) increase in log ALT levels. In the crude model, PM_10_ was not significantly associated with the log AST level (*p* = 0.4743) and NO_2_ was associated with a reduction in the log AST level (β = −0.0214; 95% CI = −0.0274, −0.0155). In all three adjusted models (Model 1–Model 3), all four air pollutants showed a statistically significant association with an increase in log AST levels (all *p* < 0.001).

The results showing the association of ambient air pollution with liver enzymes for drinkers and nondrinkers are presented in Table 4. There was no considerable difference in the effect of NO_2_ and SO_2_ on liver enzymes for the drinkers and non-drinkers, but the PM_10_ and CO showed distinct differences between the two groups. In non-drinkers, PM_10_ and CO were not statistically related to liver enzyme levels, but in drinkers, exposure to these air pollutants was significantly associated with elevated log ALT and log AST levels. In the drinkers, an IQR (9 μg/m^3^) increase in PM_10_ concentration was associated with increases of 0.0285 IU/L (95% CI = 0.0201, 0.0368) in the log ALT level and 0.0139 IU/L (95% CI = 0.0079, 0.0198) in the log AST level. In non-drinkers, an IQR (0.1 ppm) increase in CO concentration in the drinking group showed an increase of 0.0247 IU/L (95% CI = 0.0182, 0.0311) and 0.0164 IU/L (95% CI = 0.0118, 0.0210) in log ALT and log AST, respectively.

**Figure 1:**
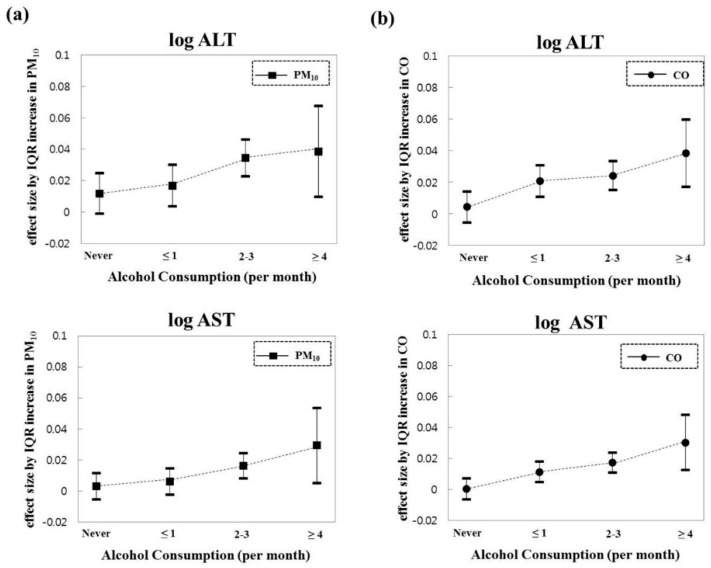
The beta coefficients and 95% confidence intervals in liver enzyme levels per IQR increases in annual average (**a**) PM_10_ and (**b**) CO according to the monthly frequency of alcohol consumption.

In Table 4, differences in the association between air pollution and liver enzyme levels according to the alcohol drinking status, especially in PM_10_ and CO, are demonstrated. The effects of PM_10_ and CO on liver enzymes through stratification of alcohol consumption level has also been identified, as shown in Figure 1. The effect size of PM_10_ and CO on log ALT and log AST levels gradually increased along with the monthly frequency of alcohol consumption.

## 4. Discussions

This study investigated the relationships between exposure to ambient air pollution and liver enzyme (ALT and AST) levels in Korean adults. In addition, the modification effects of alcohol drinking in these associations were evaluated. When adjusting for all covariates (Model 3), we observed significant associations between annual average concentrations of the air pollutants (PM_10_, NO_2_, SO_2_, and CO) and liver enzyme levels (all *p* < 0.001). The associations with liver enzymes, especially in exposure to PM_10_ and CO exposures, were modified by alcohol drinking status. The effects on ALT and AST levels gradually increased with the frequency of alcohol consumption.

Previous studies have found relationships between short-term and long-term exposure to ambient air pollution and liver enzyme levels such as ALT, AST, and GGT [12,15,22]. In a 2013 study of 5,892 adults in southern Germany, Markevych et al. identified a strong effect of PM_2.5_ on elevated GGT level as well as an effect on patients with CVD [12]. This study did not find any differential effects in patients with CVD (data not shown). A panel data analysis of elderly Koreans aged ≥60 years reported that short-term exposure to ambient air pollutants, including PM_2.5_, NO_2_, and ozone (O_3_), was closely related to elevated liver enzyme levels [15]. It also found that the negative short-term effect of PM_2.5_ on liver enzymes could be controlled by regular exercise or reduced alcohol consumption. In light of our findings, intervention for alcohol abstinence is necessary not only for the elderly but also for the general adult population, in order to prevent the increase of liver enzymes caused by air pollution. A study on the effect of long-term exposure to environmental pollution [22] found that liver enzyme levels were 0.8–2.8 times higher in 46 subjects living near an oil drilling site than in 61 subjects living in a control site. The results suggested that long-term exposure to environmental pollutants may be involved in liver abnormality or injury. Our study was unable to assess the effect of PM_2.5_ on liver enzymes because of the absence of data, but the findings that exposure to air pollution has harmful effects on liver enzymes were consistent with previous reports.

The biological mechanisms underlying the harmful effects of ambient air pollution on the liver have not yet been clarified but may be explained by several direct or indirect causes. One of the most plausible mechanisms is the oxidative stress response. Oxidative stress is known to contribute to liver injury and disease pathogenesis [11,23,24]. The liver is a major organ affected by reactive oxygen species (ROS). Excess production of ROS disturbs redox homeostasis in the liver and increases oxidative stress. Exposure to ambient air particles and excessive alcohol consumption activate ROS production [25,26] and increase systemic oxidative stress that can affect liver homeostasis [11,27]. That is to say that both environmental pollution and alcohol consumption are closely related to increased oxidative stress, and they may have a synergistic impact on liver enzymes. Systemic inflammation also contributes to liver damage and affects homeostasis [28]. Air pollutants may influence liver dysfunction via direct and indirect inflammatory responses. Ambient airborne PM activates immune cells, such as Kupffer cells and hepatic macrophages, thereby stimulating the secretion of pro-inflammatory cytokines, such as interleukin (IL)-6 and IL-β [29,30]. This inflammation pathway may occur with the translocation of PM from the lungs to the liver [29]. Inhaled PM stimulates an initial immune response by contacting the immune cells, such as the alveolar or bronchial macrophages, that release inflammatory cytokines, such as IL-6, IL-8, and macrophage inflammatory protein-1, into the bloodstream. Chronic alcohol use is also associated with inflammatory responses, such as those caused by PM exposure [31,32]. The pro-inflammatory environment may contribute to the development or progression of various liver diseases.

Most previous studies have focused on acute or chronic effects of pollutants in restricted regions or specific age groups, especially the elderly. The present large-scale study evaluated nationwide data from the general adult population of South Korea to identify the association between air pollution and liver enzymes. To the best of our knowledge, this is the first study of this type, and the results indicate that annual average concentrations of ambient air pollutants were significantly associated with liver enzyme levels and this association was enhanced by alcohol consumption.

This study was limited by its cross-sectional design, which does not allow for determination of causal relationships for the observed effects of alcohol consumption and ambient air pollution on liver enzymes. Second, we could not assess the impact of PM_2.5_, which might penetrate more deeply into the liver, lungs, or other organs than other air pollutants, because of the lack of data. Third, we could not measure an accurate exposure level to ambient air pollution in each individual, because of the lack of relevant information about air pollution at workplaces, residential periods, and the distance from the residence to major roads. In addition, our assessment method for air pollution exposure results in the same exposure for multiple subjects in the same admin district. This means that intra-city variability in air pollutant concentrations cannot be examined. Furthermore, it was difficult to estimate a longer time interval of exposure concentration, because we do not have any information on the residential history of the participants. Therefore, this study only used the annual average air pollutant concentrations of the participant’s survey year. Lastly, we did not consider various factors that might impact liver functions such as intake of medicine, heavy metals exposure, sleep time, and stress status.

## 5. Conclusions

This study identified that chronic exposures to ambient air pollutants were significantly associated with elevated liver enzyme levels using large-scale nationwide data from South Korea. There was evidence for a synergistic effect of alcohol consumption and chronic exposure to ambient air pollution on liver enzyme levels (ALT and AST) in the general adult population.

## Figures and Tables

**Table 1:** Characteristics of the study subjects (*n* = 36,151).

Characteristics	Mean ± SD or *n* (%)	Log ALT *p*-Value	Log AST *p*-Value
Age (years)	49.5 ± 16.0	<0.0001	<0.0001
Female	20,672 (57.2)	<0.0001	<0.0001
Education level		<0.0001	<0.0001
≤Elementary school	9367 (25.9)		
Middle school	3964 (11.0)		
High school	12,259 (33.9)		
≥College or graduate school	10,561 (29.2)		
Residential region		0.0010	<0.0001
Urban	24,302 (67.2)		
Rural	11,849 (32.8)		
Smoking		<0.0001	<0.0001
Current smokers	7748 (21.4)		
Former smokers	7193 (19.9)		
Never	21,210 (58.7)		
Alcohol Consumption (freq. per month)		<0.0001	<0.0001
Never	10,230 (28.3)		
≤1	10,363 (28.7)		
2–3	12,977 (35.9)		
≥4	2581 (7.1)		
Moderate physical activity		<0.0001	0.0008
Yes	14,162 (39.2)		
No	21,989 (60.8)		
Stress		0.4000	0.0003
Yes	9676 (26.8)		
No	26,475 (73.2)		
BMI (kg/m^2^)	23.7 ± 3.3	<0.0001	<0.0001
WC (cm)	81.3 ± 9.9	<0.0001	<0.0001
SBP	118.8 ± 17.4	<0.0001	<0.0001
Hypertension	11,010 (30.5)	<0.0001	<0.0001
TC (mg/dL)	188.6 ± 36.2	<0.0001	<0.0001
FBG (mg/dL)	98.0 ± 22.6	<0.0001	<0.0001
T2D	3487 (9.7)	<0.0001	<0.0001
CVD	1518 (4.2)	<0.0001	<0.0001
Liver enzymes (IU/L)			
ALT	21.7 ± 18.9		
AST	22.5 ± 13.4		

SD, standard deviation; BMI, body mass index; WC, waist circumference; SBP, systolic blood pressure; TC, total cholesterol; FBG, fasting blood glucose; T2D, type 2 diabetes, CVD, cardiovascular disease; ALT, alanine aminotransferase; AST, aspartate aminotransferase.

**Table 2:** Distribution of air pollutants (annual average concentrations).

Air Pollutants	Urban		Rural		Total	
	Mean (Median)	IQR	Mean (Median)	IQR	Mean (Median)	IQR
PM_10_ (μg/m^3^)	51.7 (51)	10	48.9 (48)	7	50.7 (49)	9
NO_2_ (ppb)	28.5 (30)	7	18.0 (18)	3	25.1 (24)	11
SO_2_ (ppb)	5.5 (5)	1	5.2 (5)	2	5.4 (5)	1
CO (ppm)	0.6 (0.6)	0.1	0.5 (0.5)	0.1	0.5 (0.6)	0.1

IQR, interquartile range; PM_10_, particulate matter <10 μm in diameter; NO_2_, nitrogen dioxide; SO_2_, sulfur dioxide; CO, carbon monoxide.

**Table 3:** Regression results for liver enzyme levels per IQR increases in annual average air pollution.

Air Pollution	Crude		Model 1		Model 2		Model 3	
	β (95% CI)	*p*-Value	β (95% CI)	*p*-Value	β (95% CI)	*p*-Value	β (95% CI)	*p*-Value
**log ALT**								
PM_10_ (μg/m^3^)	0.0183 (0.0104, 0.0262)	<0.0001	0.0216 (0.0140, 0.0292)	<0.0001	0.0207 (0.0131, 0.0283)	<0.0001	0.0228 (0.0157, 0.0299)	<0.0001
NO_2_ (ppb)	−0.0066 (−0.0156, 0.0025)	0.1547	0.0135 (0.0007, 0.0263)	0.0387	0.0127 (−0.0001, 0.0254)	0.0520	0.0251 (0.0132, 0.0371)	<0.0001
SO_2_ (ppb)	0.0116 (0.0065, 0.0167)	<0.0001	0.0128 (0.0079, 0.0176)	<0.0001	0.0130 (0.0081, 0.0178)	<0.0001	0.0132 (0.0087, 0.0177)	<0.0001
CO (ppm)	0.0136 (0.0073, 0.0198)	<0.0001	0.0140 (0.0081, 0.0198)	<0.0001	0.0134 (0.0075, 0.0193)	<0.0001	0.0165 (0.0110, 0.0219)	<0.0001
**log AST**								
PM_10_ (μg/m^3^)	0.0019 (-0.0033, 0.0071)	0.4743	0.0098 (0.0048, 0.0148)	0.0001	0.0098 (0.0048, 0.0148)	0.0001	0.0105 (0.0056, 0.0154)	<0.0001
NO_2_ (ppb)	−0.0214 (−0.0274, −0.0155)	<0.0001	0.0131 (0.0047, 0.0215)	0.0022	0.0131 (0.0047, 0.0214)	0.0021	0.0157 (0.0074, 0.0239)	0.0002
SO_2_ (ppb)	0.0057 (0.0023, 0.0091)	0.0008	0.0073 (0.0041, 0.0105)	<0.0001	0.0074 (0.0042, 0.0105)	<0.0001	0.0073 (0.0042, 0.0104)	<0.0001
CO (ppm)	0.0066 (0.0025, 0.0107)	0.0017	0.0096 (0.0057, 0.0135)	<0.0001	0.0099 (0.0060, 0.0137)	<0.0001	0.0115 (0.0077, 0.0153)	<0.0001

CI, confidence interval; ALT, alanine aminotransferase; AST, aspartate aminotransferase; PM_10_, particulate matter <10 μm in diameter; NO_2_, nitrogen dioxide; SO_2_, sulfur dioxide; CO, carbon monoxide. The beta coefficients (95% confidence interval) and p-values were calculated with natural log-transformed values of ALT and AST. The beta coefficients (95% confidence interval) in each air pollutant were scaled to the interquartile range for each pollutant (9 μg/m^3^ for PM_10_, 11 ppb for NO_2_, 1 ppb for SO_2_, and 0.1 ppm for CO, respectively). Model 1 was adjusted for demographic variables including age, sex, education level, household income, and urbanity. Model 2 was adjusted for demographic variables plus health-related lifestyle including smoking status, alcohol drinking, moderate physical activity, and usual stress status. Model 3 was adjusted for demographic variables and health-related lifestyle plus clinical variables including body mass index, waist circumference, total cholesterol, systolic blood pressure, hypertension, diabetes, and cardiovascular disease.

**Table 4:** Regression results for liver enzyme levels per IQR increases in annual average air pollution according to the alcohol drinking status.

Air Pollution	Non-Drinker (n = 10,230)		Drinker (n = 25,921)	
	β (95% CI)	*p*-Value	β (95% CI)	*p*-Value
**log ALT**				
PM_10_ (μg/m^3^)	0.0119 (−0.0009, 0.0248)	0.0693	0.0285 (0.0201, 0.0368)	<0.0001
NO_2_ (ppb)	0.0266 (0.0040, 0.0492)	0.0209	0.0249 (0.0109, 0.0388)	0.0005
SO_2_ (ppb)	0.0137 (0.0055, 0.0220)	0.0011	0.0121 (0.0068, 0.0174)	<0.0001
CO (ppm)	0.0043 (−0.0056, 0.0142)	0.3984	0.0247 (0.0182, 0.0311)	<0.0001
				
**log AST**				
PM_10_ (μg/m^3^)	0.0030 (0.0056, 0.0116)	0.4945	0.0139 (0.0079, 0.0198)	<0.0001
NO_2_ (ppb)	0.0159 (−0.0007, 0.0310)	0.0403	0.0165 (0.0066, 0.0264)	0.0011
SO_2_ (ppb)	0.0070 (0.0015, 0.0126)	0.0132	0.0073 (0.0035, 0.0110)	0.0002
CO (ppm)	0.0003 (−0.0063, 0.0070)	0.9217	0.0164 (0.0118, 0.0210)	<0.0001

CI, confidence interval; ALT, alanine aminotransferase; AST, aspartate aminotransferase; PM_10_, particulate matter <10 μm in diameter; NO_2_, nitrogen dioxide; SO_2_, sulfur dioxide; CO, carbon monoxide. The beta coefficients (95% confidence interval) and p-value were calculated with natural log-transformed values of ALT and AST. The beta coefficients (95% confidence interval) in each air pollutant were scaled to the interquartile range for each pollutant, respectively (9 μg/m^3^ for PM_10_, 11 ppb for NO_2_, 1 ppb for SO_2_, and 0.1 ppm for CO). The association results were adjusted for age, sex, education level, household income, urbanity, smoking status, moderate physical activity, stress status, body mass index, waist circumference, total cholesterol, systolic blood pressure, hypertension, diabetes, and cardiovascular disease.
